# Post-CMOS processing challenges and design developments of CMOS-MEMS microheaters for local CNT synthesis

**DOI:** 10.1038/s41378-023-00598-w

**Published:** 2023-11-06

**Authors:** Avisek Roy, Bao Q. Ta, Mehdi Azadmehr, Knut E. Aasmundtveit

**Affiliations:** Department of Microsystems, University of South-Eastern Norway, 3184 Horten, Norway

**Keywords:** Carbon nanotubes and fullerenes, Other nanotechnology

## Abstract

Carbon nanotubes (CNTs) can be locally grown on custom-designed CMOS microheaters by a thermal chemical vapour deposition (CVD) process to utilize the sensing capabilities of CNTs in emerging micro- and nanotechnology applications. For such a direct CMOS-CNT integration, a key requirement is the development of necessary post-processing steps on CMOS chips for fabricating CMOS-MEMS polysilicon heaters that can locally generate the required CNT synthesis temperatures (~650–900 °C). In our post-CMOS processing, a subtractive fabrication technique is used for micromachining the polysilicon heaters, where the passivation layers in CMOS are used as masks to protect the electronics. For dielectric etching, it is necessary to achieve high selectivity, uniform etching and a good etch rate to fully expose the polysilicon layers without causing damage. We achieved successful post-CMOS processing by developing two-step reactive ion etching (RIE) of the SiO_2_ dielectric layer and making design improvements to a second-generation CMOS chip. After the dry etching process, CMOS-MEMS microheaters are partially suspended by SiO_2_ wet etching with minimum damage to the exposed aluminium layers, to obtain high thermal isolation. The fabricated microheaters are then successfully utilized for synthesizing CNTs by a local thermal CVD process. The CMOS post-processing challenges and design aspects to fabricate CMOS-MEMS polysilicon microheaters for such high-temperature applications are detailed in this article. Our developed process for heterogeneous monolithic integration of CMOS-CNT shows promise for wafer-level manufacturing of CNT-based sensors by incorporating additional steps in an already existing foundry CMOS process.

## Introduction

The integration of nanomaterials into microsystems enables a medium of sensing various gases^1–3^, bioagents^4,5^, chemicals^6^, environmental contaminants^7^, etc. based on the change in different properties of the material. Carbon nanotubes (CNTs) are an excellent example of nanomaterials with exceptional electrical^8^, electronic^9^, optoelectronic^10^, thermal^11^ and nanomechanical^12^ properties. Their properties can be utilized in diverse smart applications^13–17^. CNTs have a high surface area in proportion to their volume, which is particularly effective for gas sensing applications^18–20^.

There are various methods of integrating CNTs in a microsystem^21–24^. These methods can be divided into two categories: direct CNT growth on the microstructures or depositing already synthesized CNTs on them. The latter method mainly involves wet nanomaterial deposition techniques such as dielectrophoresis (DEP). In addition to surface contamination during the wet process, poor bonding between the nanomaterials and microstructures is another issue associated with wet methods^25^. Moreover, the lack of alignment along with the nonuniform deposition of CNTs^22^ are major obstacles preventing the realization of a reliable and repeatable integration process in most of these wet deposition techniques. The approach of directly synthesizing CNTs on microstructures can overcome these challenges due to the controlled growth method of well-aligned CNTs at desired locations. However, the CNT synthesis temperature needed at the growth locations can be very high (~650–900 °C)^26^.

CNTs can be directly synthesized on a microelectromechanical system (MEMS) by local resistive heating of the growth structure^27–29^. The sensing capabilities of such CNTs are conveniently demonstrated by MEMS-CNT integration^30,31^, but electronic circuits also need to be integrated for commercially manufacturing compact CNT-based smart sensors. Direct integration of CNTs in complementary metal-oxide-semiconductor (CMOS) chips shows the potential for realizing such sensors, which will have a native capability of processing electrical signals. However, a CMOS-compatible temperature (~300–400 °C)^32,33^ must be ensured during the CNT synthesis process on a CMOS structure. Suspended microstructures are therefore ideal for generating a high CNT growth temperature by Joule heating due to isolating the heat. This makes such structures suitable to manufacture in a standard MEMS fabrication process. Therefore, forming MEMS structures in CMOS chips can lead to an effective method of direct CMOS-CNT integration. CNTs have been synthesized in CMOS^34,35^ with extensive post-processing steps, which we propose to reduce to achieve a potential wafer-level compatible approach that can be incorporated in a commercial CMOS foundry with less process development.

There are numerous approaches for integrating MEMS and CMOS, which can mostly be classified into hybrid multichip and system-on-chip (SoC) integration solutions^36^. Hybrid multichip integration approaches, such as system-in-package (SiP), refer to the process where MEMS and CMOS chips are manufactured separately in their dedicated processes before heterogeneous integration. In SiP, CNTs can be synthesized on MEMS chips and vertically stacked on chips containing ASICs to avoid direct CNT growth on CMOS. On the other hand, MEMS and CMOS structures are fabricated on the same substrate in SoC integration processes. SoC solutions are compact in size, cost-effective due to possible wafer-level packaging with low testing costs and provide high integration densities; however, the fabrication processes of these techniques can be challenging because of high complexity and low flexibility^36^. Among the SoC solutions, monolithic integration of CMOS MEMS can be classified into pre-CMOS, intra-CMOS and post-CMOS approaches based on the formation order of the MEMS^37^. The latter approach is also known as CMOS-MEMS and offers advantages over the other two approaches based on CMOS compatibility, manufacturing costs and flexibility in design due to the convenience of choosing appropriate CMOS technology and foundry^36,37^. Therefore, we selected the CMOS-MEMS technique to fabricate microheaters in industrially manufactured CMOS chips for local CNT synthesis.

**Fig. 1:**
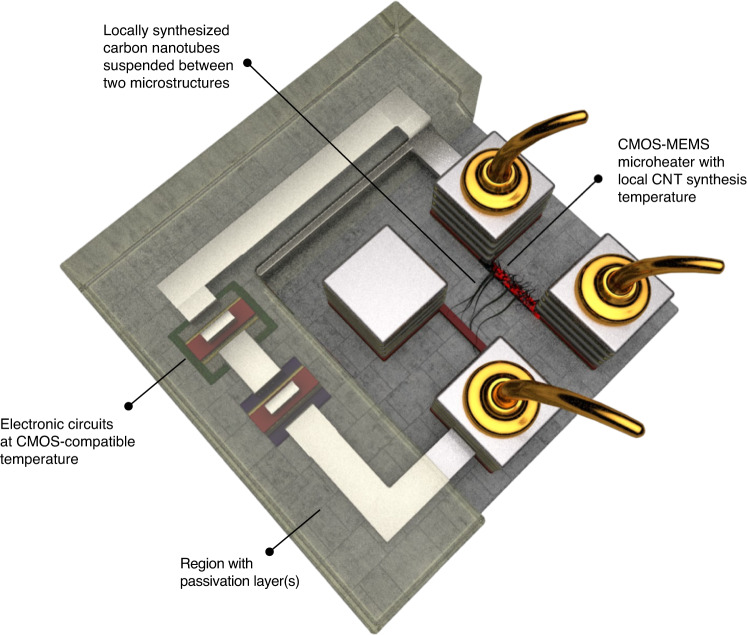
Concept of local CNT synthesis on a CMOS-MEMS microheater at high temperatures with ICs at low ambient temperature

CMOS-MEMS structures are fabricated by necessary post-processing of the CMOS chips. A standard CMOS chip has polysilicon layers for constructing gates of the transistors and metal layers for routing electrical connections, both of which can be used as microheaters. However, based on the material properties suitable for efficient resistive heating along with the temperature requirements in CNT synthesis, polysilicon is the better material option for CMOS-MEMS microheaters^38^. These polysilicon microheaters can be designed with appropriate lengths and widths; however, the thicknesses will be CMOS process dependent. The polysilicon layers in CMOS are covered with dielectric and passivation layers, which need to be selectively removed by the subtractive CMOS-MEMS microfabrication approach^37^ to make the polysilicon microheaters accessible for growing CNTs over them. The polysilicon CMOS-MEMS microheaters can be fully or partially released by underetching the dielectric layer beneath them. This concept is illustrated in Fig. 1, where the exposed polysilicon microheater is heated through the wire-bonded contact pads, while the CMOS circuits stay in a CMOS-compatible region. CNTs are locally synthesized on the CMOS-MEMS microheater by a thermal chemical vapour deposition (CVD) process, where the chamber remains at room temperature. Using a local electric field, some synthesized CNTs can be directed towards an adjacent microstructure to establish connections. The connected CNTs can be used for gas sensing due to their change in resistivity upon exposure to various gases. Our group has previously explored the electrical properties of locally grown CNTs on a MEMS platform and validated the gas sensing process using this approach^31,39^.

Many challenges may arise during the post-processing of the CMOS chips in the selected SoC CMOS-MEMS integration method. Although this integration solution has several mentioned benefits, the product development is slower than typically development cycles due to the involved complexity and low flexibility^36^. We designed and fabricated two generations of CMOS chips in an industrial CMOS process to grow CNTs on CMOS-MEMS heaters. Based on the post-processing results of the previous generation CMOS chips, design improvements were made on the succeeding generation of CMOS chips, and post-processing approaches were modified accordingly. We found that proper design of the microstructures plays a very important role in realizing CMOS-MEMS structures along with the selection of appropriate post-processing approaches. In this paper, we present the encountered post-processing challenges and the incorporated design upgrades in CMOS chips to successfully fabricate CMOS-MEMS microheaters for local CNT synthesis.

## Results and Discussion

### Polysilicon microheater design in CMOS

**Fig. 2:**
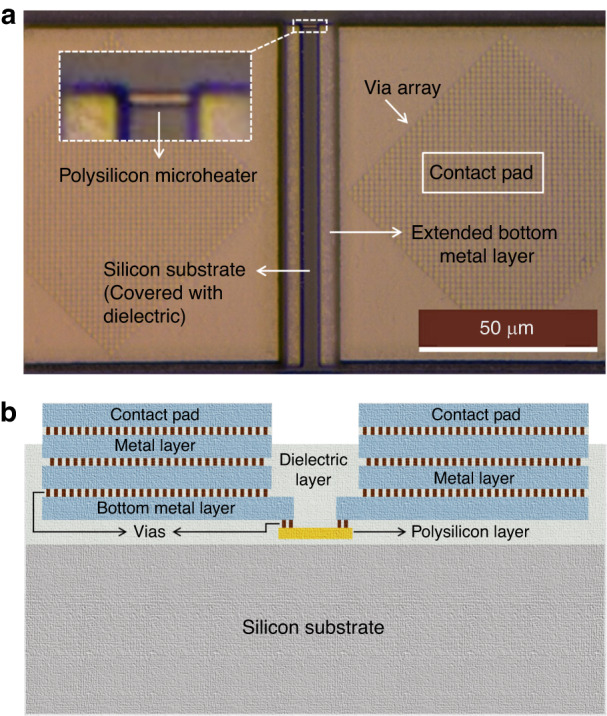
A typical polysilicon microheater from the first-generation design. **a** Optical micrograph and **b** cross-sectional illustration

A CMOS chip is designed and fabricated in a standard AMS 350 nm process. The CMOS process includes two polysilicon layers, poly-1 and poly-2. The thickness of the polysilicon layers is ~200–300 nm^40^. The poly-2 layer is ~1.5 times thinner than poly-1 and has ~4 times higher electrical resistivity at room temperature (measured to ~11 Ω.µm), which is beneficial for the design of microheaters. For the local synthesis of CNTs, various microstructures of different shapes were designed using the two polysilicon layers. The polysilicon microstructures are connected to custom-designed contact pads made of top interconnecting aluminium layers for electrical connection. Figure 2a shows a design containing a polysilicon microheater and associated contact pads. In the microheater designs, the objective was to make polysilicon resistors that can efficiently generate CNT synthesis temperature by local resistive heating at low power and with minimum heat loss. A reduction in the microheater surface area is beneficial in limiting conductive heat loss. Therefore, the widths of the designed polysilicon heaters were mostly kept within the range of 0.7 µm to 1 µm, with 0.7 µm being the lowest valid dimension for poly layers in the used CMOS technology. For efficient heating, a designed microheater should have the dominant resistance within the Joule heating circuitry. Considering the boundary conditions, the microheaters were designed within a varied length ranging mostly from 6 µm to 15 µm. Heater dimension considerations have previously been analysed based on simulation results during a feasibility study of local CNT synthesis on CMOS^41,42^. The thermomechanical simulations of polysilicon microstructures also showed that high CNT growth temperatures can be achieved on CMOS polysilicon microheaters while maintaining CMOS-compatible temperatures (<300 °C) on the chip. In addition, we characterized some of the microheaters under an infrared microscope, which showed ~30 °C temperature around the chip surface when an on-chip aluminium heater was operated at its melting temperature (~660 °C)^41^. A minimum distance of 15 µm is assigned between two contact pads for the ease of wire bonding. To ensure this distance, only the bottom metal layer is extended to establish a connection with the poly layer, as shown in Fig. 2a.

A cross-section illustrated in Fig. 2b shows all the layers in the CMOS microheater design. The heater is surrounded by silicon dioxide (SiO_2_) dielectric layers and connected with the extended bottom metal layer through a few vias. This AMS CMOS process consists of four interconnecting metal layers. The top contact pad connects to the bottom metal layer through two more metal layers and corresponding arrays of vias. The surface of the dielectric layer is normally covered with passivation/protection layers in typical CMOS chips. Our chips need post-processing steps to expose the polysilicon layers for CNT synthesis. Therefore, through the selective placement of a design layer, the passivation layers are avoided over the microheaters. In this way, the passivation layers are only removed on the indicated regions during the CMOS fabrication process, while the regions over the CMOS circuits are still protected by the passivation layers. As a result, we can avoid several post-processing steps needed for selective removal of the passivation layers.

### Fabrication steps and requirements for CMOS-MEMS heaters

**Fig. 3:**
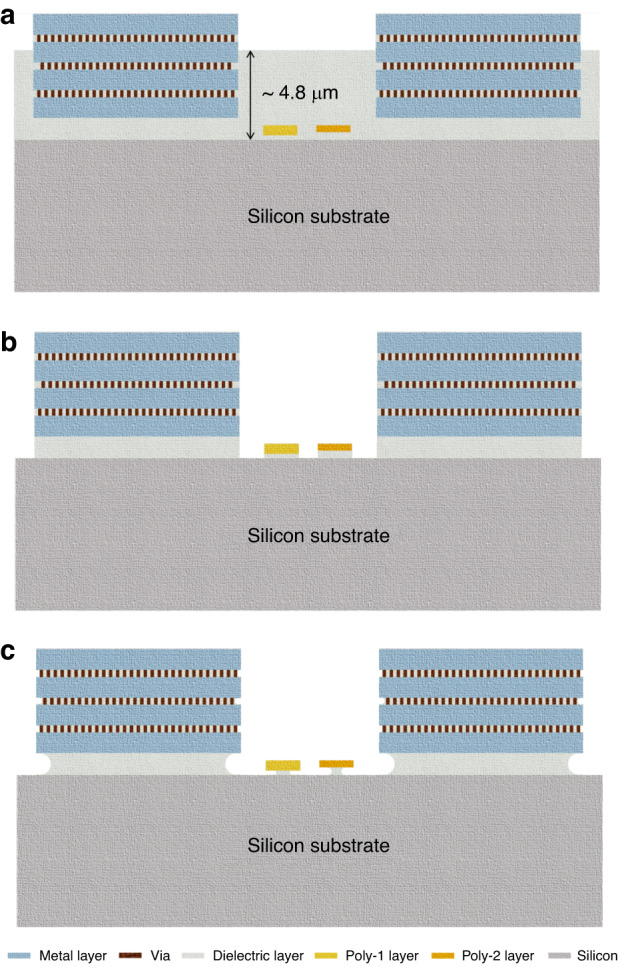
Required steps of post-CMOS processing for realizing a CMOS-MEMS polysilicon microheater. **a** Initial view after receiving the chips from the CMOS foundry, **b** dry etching of the exposed dielectric layer, and **c** wet etching for a limited duration to partially or fully suspend the microheaters

In the subtractive post-CMOS processing technique, the thick dielectric layer needs to be removed to realize CMOS-MEMS microstructures. The cross-sectional illustrations in Fig. 3 indicate the steps of the process. The polysilicon microstructures are exposed by etching the SiO_2_ layer. In this dielectric etching process, it is essential that the selectivity between SiO_2_ and polysilicon is very high to keep the polysilicon structures intact. For SiO_2_ etching, buffered oxide etch (BOE) is commonly used. Although this wet etching process provides high selectivity between SiO_2_ and polysilicon, it highly affects the aluminium metal layer. Additionally, a wet etching process risks contaminating the delicate CMOS chip surface. Therefore, a highly selective dry etching process is more suitable. During dry etching, the substrate temperature of the sample holder must not exceed the CMOS-compatible temperature.

It is also important to maintain CMOS-compatible temperatures during the CNT synthesis process. Therefore, the high temperature on the microheater during the CNT growth process should be highly localized. Such CMOS microheaters can be partially or fully suspended by etching the dielectric layer underneath the heaters to reduce or remove the heat conduction path from the heater to the Si bulk. As the width of the microheaters is within 1 µm, dry etching can suffice to partially release the heaters unless the etching process is completely anisotropic. To fully release the microheaters, a time-controlled wet etching process can be performed, where the risk of stiction should be considered.

### Post-CMOS processing challenges and limitations in first-generation designs

**Fig. 4:**
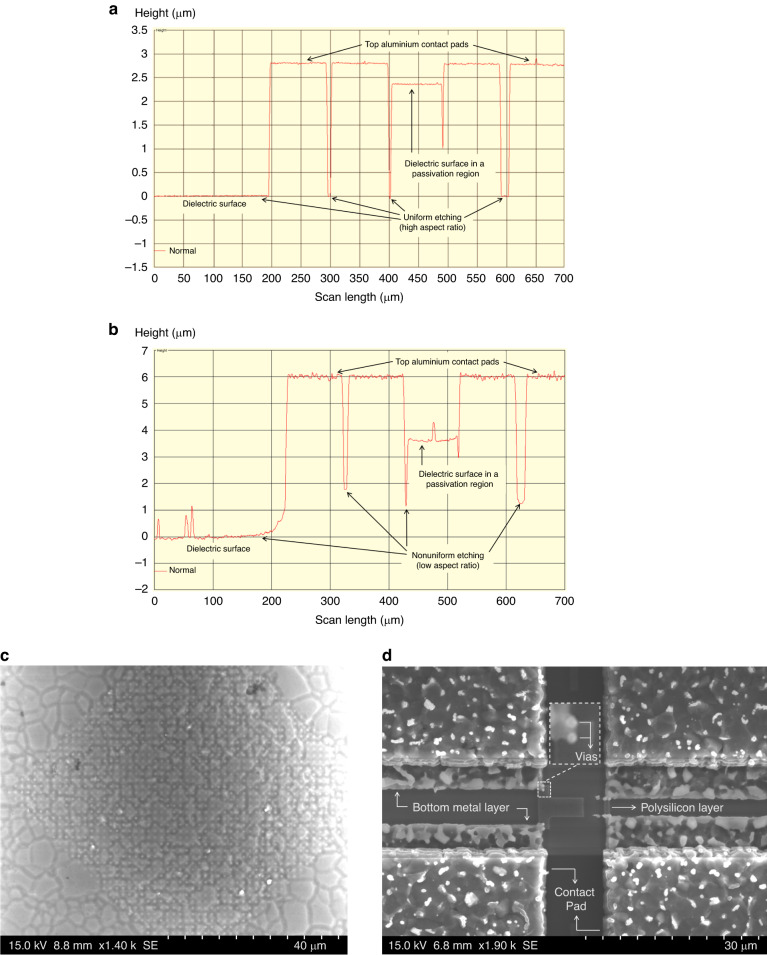
Chip surface characterization results after dielectric etching with DER-1 or DER-2. Etching profile after **a** DRIE using DER-1 and **b** RIE with ICP source using DER-2 recipe. Damage to aluminium layers after RIE with high ICP power: **c** surface of a contact pad and **d** possible disconnections of the extended bottom metal layers and vias that link the polysilicon heaters

To expose the polysilicon layers, etching of an ~4.2 µm thick SiO_2_ layer is needed. As the opening over the microheater region is small in the first-generation chip designs, deep reactive ion etching (DRIE) was used as an initial approach for dielectric etching. The recipe used for SiO_2_ DRIE (named dry etching recipe, DER-1) provided a high etch rate and high aspect ratio. The etching profile, as obtained by profilometry, in Fig. 4a shows that the etched depth in the regions of small openings was similar to that in the regions with large openings. However, the selectivity of DER-1 was very poor, resulting in significant etching of the polysilicon layers after exposure. Therefore, a new SiO_2_ etching recipe (DER-2) is used in a reactive ion etching (RIE) system with an inductively coupled plasma (ICP) source, which facilitates a wider range of gases. Details of all the applied etching recipes are provided in the *Materials and Methods* section.

Etching with DER-2 ensured a decent selectivity (~1:2) between polysilicon and SiO_2_; however, it also resulted in nonuniform etching due to the low aspect ratio. The surface profile in Fig. 4b shows the lower etching depth in the regions with small openings. The passivation layer was etched at a slower rate in both recipes, resulting in a thick dielectric in those regions, as shown in Fig. 4a, b. We also notice that the surface roughness of the structures in Fig. 4b is higher than observed in the results of Fig. 4a. The temperature of the sample holding substrate was not controllable in the RIE system used; hence, the samples were at an elevated temperature during the RIE process, which may explain the cause of the higher surface roughness. The introduction of a cooling cycle (using argon) between short etching cycles helped improve the surface profile.

It is important to achieve a more uniform etching profile. As the opening areas over different microheater designs are not the same, a variable etching depth occurs due to a lower aspect ratio, which exposes some microheaters earlier than others. As a result, if the etching duration is increased to expose heaters with smaller opening areas, the already exposed heaters will be at risk of being etched because the etching selectivity is not extremely high. The issue of a low aspect ratio is also relevant for microheater characterization. It is challenging to identify whether the microheaters are exposed. An optical method of characterization (such as interferometry) is not applicable as the heaters are covered by transparent SiO_2_. Due to the small opening area and dimensions of the heaters, measuring with a profilometer is challenging, and the measurements are also limited by the diameter of the probe tip. Observing cross-sections is an effective method for this characterization, but this destructive method is not reasonable in our case due to the limited number of samples (CMOS chips). A suitable method is using a probe station, where the microprobes can be placed on the sample to measure conductivity. A conductive surface means that SiO_2_ is removed, and polysilicon or bulk silicon is exposed in the measured region. This method can only be used in areas with larger openings, which also signifies that a higher aspect ratio is needed during SiO_2_ etching. With equal etching depths for small and large openings (similar to Fig. 4a), confirming a conductive surface on a larger region will also ensure exposure of the microheaters.

Initially, the chips were etched without enabling the ICP source. After providing it some power, a higher aspect ratio is obtained. Although very narrow opening areas still had residues, an opening window wider than 35 µm had adequate etching. Higher ICP power indeed helps improve the aspect ratio; however, increasing it above 200 W resulted in a higher substrate temperature during the ICP-RIE process. As a result, the exposed aluminium surfaces were highly damaged (Fig. 4c, d) due to enhanced physical etching at high temperatures.

Damaged aluminium contact pads (Fig. 4c) are problematic for wire bonding. Figure 4d shows that the bottom extended metal layer connected to the polysilicon microstructures is also exposed and damaged. Due to the metal damage, the vias in between the bottom metal layer and polysilicon layer are clearly visible in the magnified inset of Fig. 4d. As a consequence of this damage, the polysilicon heaters were disconnected from the bottom metal layer due to the barely connected vias. Therefore, to limit the extensive damage to the exposed aluminium layers while obtaining a high aspect ratio (uniform etching), the power of the ICP source was limited to 200 W during the ICP-RIE process. Settling for low ICP power is a shortcoming of the RIE system, which lacks a thermal management system for the necessary substrate area.

To partially release the microheaters (Fig. 3c), BOE was attempted for a short duration for isotropic etching of SiO_2_ under the heaters. This wet etching approach did not cause any stiction, but the aluminium layers were affected as the etchant also attacked the aluminium surface. This step may be avoided for microheaters with small surface areas since their heat conduction path through the dielectric is already small.

Apart from the post-processing challenges of the initially designed CMOS chips, some design limitations also became apparent during the experiments. An important one is the small amount of vias used for connecting the polysilicon heaters with the bottom metal layer. For most of the microheaters, only two vias were used to connect each side of the polysilicon resistors with the metal layer. As a result, the vias were subjected to a high current density during the Joule heating process when the required current to attain the high-temperature CNT synthesis condition exceeded the standard CMOS operation limits. During this process, the heaters were disconnected at the vias before reaching the CNT growth temperature in the majority of cases. Measuring the microheater temperature during Joule heating was another challenge. The width of the microheaters is too small to obtain reliable thermal measurements even with a high-performance mid-wave infrared (MWIR) microscope^41^ since the operating wavelength used in such microscopes is 3–5 times the width of the microheaters.

### Second-generation chip design and developments in post-CMOS processing

**Fig. 5:**
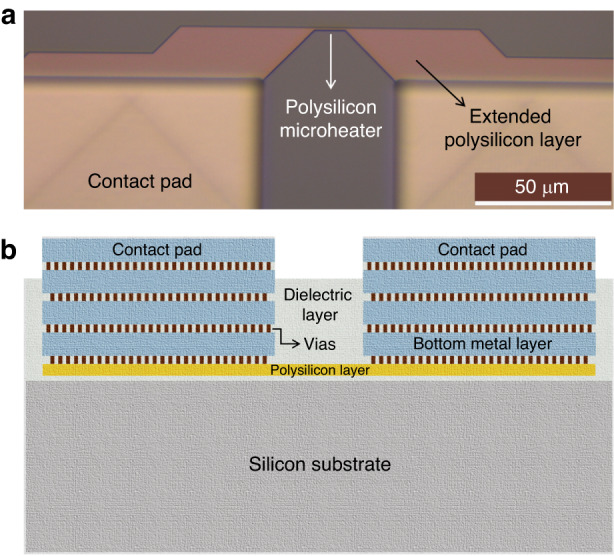
A typical polysilicon microheater from the second-generation design. **a** Optical micrograph and **b** cross-sectional illustration of a typical polysilicon microheater from the second-generation design

The challenges encountered during the post-processing of the initially designed CMOS chips revealed the necessity of design improvements for the microheaters as well as improving the post-processing approaches. Therefore, a new set of microheaters was designed and fabricated using the same AMS 350 nm CMOS technology; a micrograph of an improved design is shown in Fig. 5a. Compared to the previous design in Fig. 2a, the bottom metal extension is eliminated to avoid metal damage and consequent disconnection from the polysilicon layers, as experienced during ICP-RIE at higher powers. The purpose of the metal extension was to keep the microheater length short and maintain a certain distance between the contact pads at the same time. In this new design, the poly layer is extended instead, while the gradual shortening of the heater ensures that the effecting heating region remains in a small area at the centre of the structure.

Distances between all the contact pads are also expanded in the new designs. The previous designs showed that uniform SiO_2_ etching over the entire CMOS chip can be obtained if the minimum opening window is wider than 35 µm. Therefore, in the second-generation CMOS chips, the minimum opening area over the polysilicon microstructures surrounded by the four contact pads is set to 50 µm × 50 µm (Fig. 5a). This design improvement resolves all aspect ratio-related challenges experienced from the post-processing of the previous generation CMOS chips.

Another design improvement is increasing the number of vias connecting the bottom metal layer and polysilicon layer. The cross-sectional illustration of the new design in Fig. 5b shows that the via array is extended to the entire contact pads. Here, the polysilicon layer covers the entire surface area underneath the contact pad, and the large array of vias over that polysilicon area establishes a connection to the metal layer above. This solves the high current density issue during the Joule heating caused by the mere number of vias used in the preceding design. Finally, in the new CMOS chip design, a few large polysilicon heaters (200 µm × 30 µm) are placed to make them suitable for potential IR microscopy during Joule heating.

**Fig. 6:**
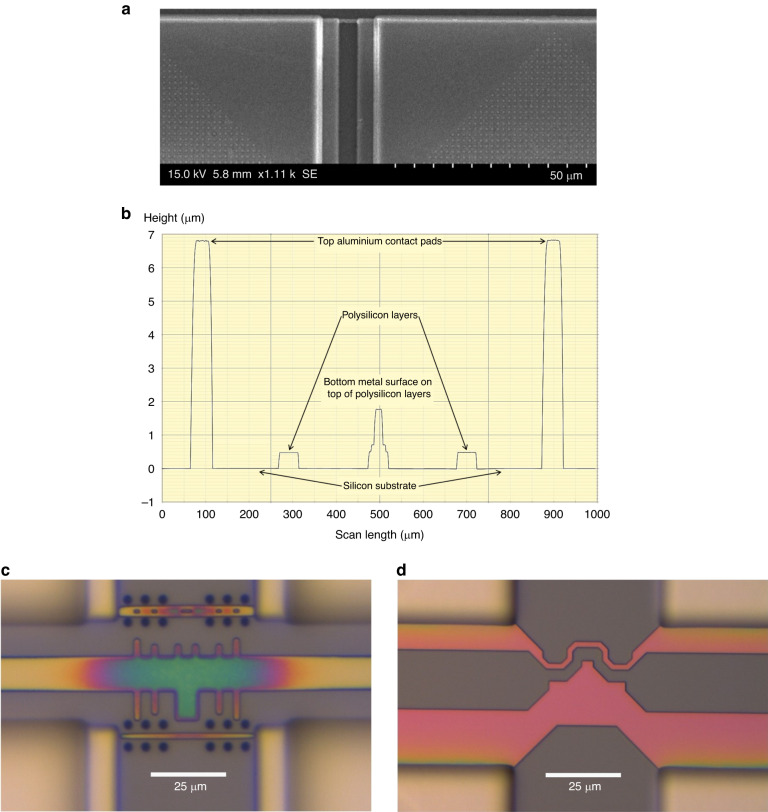
Surface characterization results of first- and second-generation CMOS chips after dielectric etching with DER-3 and DER-4. **a** Aluminium surface in a first-generation CMOS chip after dry etching with DER-3; **b** Profilometer measurement scanned across different CMOS layers in a second-generation chip. Optical micrographs of polysilicon microheaters in **c** first-generation and **d** second-generation CMOS chips after dielectric etching with DER-3 and DER-4

The post-processing approach is also improved by developing two SiO_2_ etching recipes in a new ICP-RIE system. One recipe (DER-3) uses high ICP power, while no ICP power was used in the other recipe (DER-4). DER-3 has a faster SiO_2_ etching rate and ensures a high aspect ratio for uniform etching in both small and large opening regions. As this new ICP-RIE system has excellent thermal control with the option of below 0 °C temperature on the sample holding substrate during etching, we overcame the metal damage and surface roughness issues that occurred in the previous ICP-RIE system at higher ICP powers. Figure 6a shows the surface of a first-generation design after SiO_2_ etching in the new system with DER-3, which does not show any visible damage on the metal surfaces, unlike previous results in Fig. 4c, d.

The only drawback of the new ICP-RIE recipe, DER-3, is that it exhibits somewhat low selectivity between SiO_2_ and polysilicon. Therefore, it cannot be used once the polysilicon layers are exposed, a challenge which is solved by the other newly developed recipe, DER-4. DER-4 has excellent selectivity and does not etch polysilicon layers while etching surrounding SiO_2_. As the ICP source is disabled in DER-4, the etch rate and aspect ratio are lower than those for DER-3. To obtain the benefits of both recipes without damaging the polysilicon layers, we used both recipes for their specific purposes. The SiO_2_ etching started with DER-3 and stopped before the polysilicon layer was uncovered; then, the remaining exposed SiO_2_ was etched by DER-4. DER-3 provided fast etching with a high aspect ratio for removing SiO_2_, while DER-4 ensured high selectivity for fully and safely exposing the poly layers, as shown by the surface profile in Fig. 6b. We note that the thicknesses of the polysilicon layers and the SiO_2_ layers are not consistent for the CMOS chips, and the latter can vary by several tens of nanometres. Therefore, it is important to be cautious with the etch duration when using DER-3 due to its lower selectivity. Overetching with DER-3 can result in thinner polysilicon layers after exposure, making their resistance too high for useful operation. To avoid such situations, a safe margin is considered for stopping the etch with DER-3 and continuing with DER-4. Polysilicon microheaters from first- and second-generation CMOS chips after dielectric etching with DER-3 and DER-4 are shown in Fig. 6c, d, respectively; both chips were etched together for the same duration. The multicoloured patterns on the polysilicon microstructures in Fig. 6c indicate that some dielectric layer remained in the first-generation heater, which can be due to the relatively shorter opening between the top and bottom contact pads. However, since DER-4 has high selectivity, longer etching can fully expose the polysilicon layers. The second-generation heater with a larger opening is fully exposed (Fig. 6d).

**Fig. 7:**
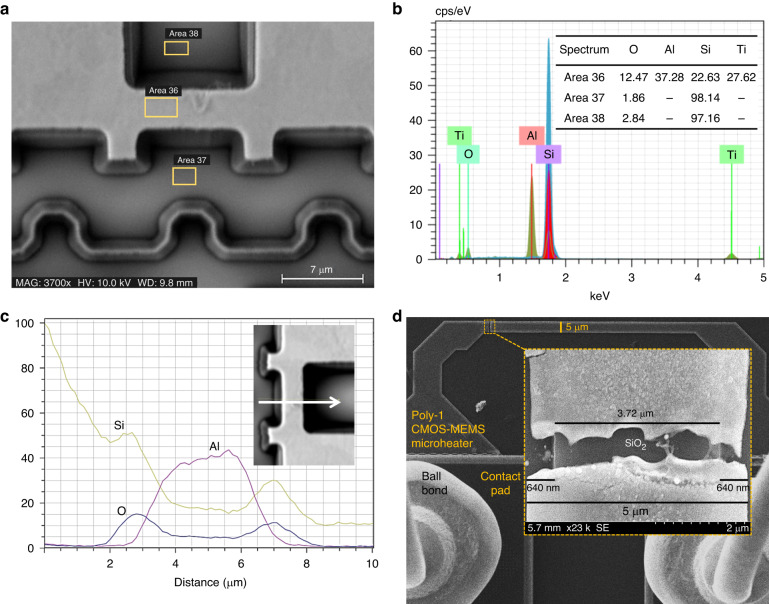
Investigation of dielectric layer (SiO_2_) underneath the CMOS metal and polysilicon layers to estimate microheater underetching after performing different etching processes on the chip. EDX analysis around a metal microstructure after dry etching: **a** electron micrograph with scanned regions; **b** EDX spectrum (elemental mapping); **c** line scan across the heater width. **d** Underetching in a poly-1 microheater after DER-4 and wet etching

EDX analysis was performed around a metal microstructure after dry etching (Fig. 7a, b) to check the anisotropy and requirement of wet etching for partial suspension of the heaters. Traces of SiO_2_ are evident underneath the top metal layer (Fig. 7b), while the line scan in Fig. 7c confirms no sign of underetching. Hence, for partial suspension of the polysilicon microheaters (Fig. 3c), we investigated SiO_2_ wet etching recipes that, unlike the BOE, do not etch aluminium. The most suitable etchant we found was Pad-etch^43,44^, which is formulated for a similar purpose of protecting aluminium pads while etching dielectric layers, as reflected by its label. Even though this etchant still mildly attacks aluminium, the damage is far less than that of BOE. We used a variant of this ‘Pad-etch’ recipe for the isotropic etching of SiO_2_. A poly-1 CMOS-MEMS microheater after exposure to dry etching followed by 30 min wet etching with pad-etch can be seen in Fig. 7d. A break in the poly-1 microheater occurred after a high-temperature Joule heating operation, which provides a view of the underneath SiO_2_ layer of the heater, as presented in the inset of Fig. 7d. An ~3.72 µm SiO_2_ layer can be found under this 5 µm wide polysilicon heater due to the ~640 nm isotropic SiO_2_ etch on each side of the heater. This confirms that the applied etching process was successful for the partial suspension of CMOS-MEMS microheaters to obtain higher thermal isolation.

### CNT synthesis on CMOS-MEMS microheater

**Fig. 8:**
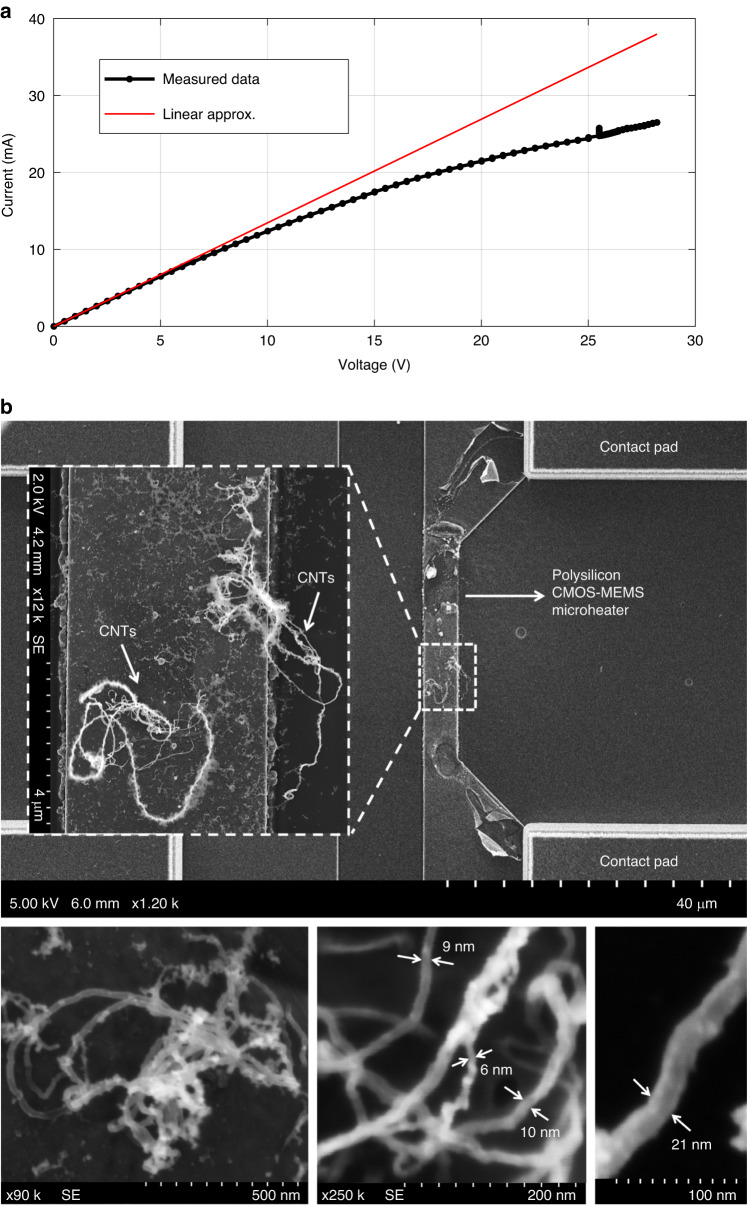
CNT synthesis on a polysilicon CMOS-MEMS microheater. **a** IV curve; **b** Synthesized CNTs

After fabricating the CMOS-MEMS polysilicon microstructures, a thin layer of iron (~2–3 nm) is deposited on the CMOS chip by electron beam (e-beam) evaporation. In this maskless process, iron deposits all over the chip; however, such a thin metal layer does not short-circuit the adjacent polysilicon structures. Iron deposited over the CMOS-MEMS microheater acts as the catalyst layer for growing CNTs. Next, the CMOS chip is attached on a chip carrier, and an electrical connection is established between the aluminium contact pads on the chip and bonding pads on the chip carrier by wire bonding. Then, the chip carrier is placed inside a custom-built CVD chamber, and electrical connections are transferred outside from the vacuum chamber by electrical feedthroughs. With suitable synthesis conditions inside the CVD chamber, the temperature of the CMOS-MEMS microheater is gradually increased by Joule heating. Figure 8a shows an I-V curve of the resistive heating process. CNT synthesis was attempted at different operating voltages of a heater to identify a suitable heater voltage range where the CNT synthesis temperature is obtained. As the heater approaches the CNT growth temperature, a carbon-containing precursor gas is introduced into the CVD chamber to initiate the CNT synthesis process. CNTs grow within 5–10 min under the synthesis conditions inside the CVD chamber, and then the heater temperature is gradually decreased to room temperature. Scanning electron microscopy (SEM) images of the synthesized CNTs on a CMOS-MEMS microheater are presented in Fig. 8b. The quality of CNTs integrated in microsystems by this technique has previously been studied by our group using the transmission mode of scanning electron microscopy or S(T)EM^45^. The results show a variety of structures while revealing defects and disorder in the synthesized CNTs. It should be noted that defective CNTs can be beneficial for gas-sensing applications^46^.

Transistors on a CNT synthesized chip have been characterized to confirm CMOS-compatibility^47^. Two on-chip PMOS transistors designed in the same AMS CMOS technology were tested before and after operating four polysilicon microheaters at CNT synthesis temperatures, where the surface area of the heaters and their distance from the transistors were varied. Operated gate-to-source voltage (V_gs_) of the transistors ranged from 0 V to –3 V, where a peak drain current (I_D_) of approximately –80 µA was found. Among 8 case studies involving 4 microheaters and 2 transistors, the I-V characteristics of the transistors after exposure to the heaters remained the same except from one scenario where the transistor closer to the heaters collapsed after using the heater with maximum surface area. We note that most of our CMOS microheaters used for the local CNT synthesis are significantly smaller than the mentioned heater with maximum surface area. Detailed results on the transistor characterizations are presented in our other work^47^.

All required post-processing has been demonstrated at the chip level; however, these processes are also scalable to the wafer level. With the obtained results, our research significantly advances progress toward our ambition of an industrially feasible wafer-level process^29^ and opens the door for a low-cost method of mass manufacturing CMOS-MEMS microheaters to locally synthesize CNTs for commercializing CNT-based sensors.

## Conclusion

In this work, we successfully synthesized CNTs on CMOS-MEMS microheaters in standard CMOS chips by a local thermal CVD process. The subtractive post-CMOS SoC integration technique was used as the cost-effective and suitable method of fabricating CMOS-MEMS microstructures for our application. The required post-processing on CMOS chips can, however, be challenging. Proper CMOS chip design and post-processing approaches hold the key to successfully realizing CMOS-MEMS microheaters for local CNT synthesis. Therefore, we designed two generations of CMOS chips, which were fabricated using commercial 350 nm CMOS technology. The previous generation chip was designed with the maximum heater efficiency in mind, in which several design limitations were revealed during post-processing. Dielectric etching is an essential step in the process to selectively expose the polysilicon layers of the CMOS chip. The challenges with dielectric etching include achieving a high aspect ratio for uniform etching with high selectivity and a good etch rate. A two-step anisotropic dielectric etching process is developed, where an ICP-RIE process is used first to attain a high aspect ratio, followed by an RIE process when selectivity becomes most important. Uniform etching is further facilitated by design improvements in the succeeding generation CMOS chip. The design and dielectric etching process are also improved to minimize damage to the metal layers and issues associated with the damage. Moreover, designs are improved in terms of maintaining proper connection between the polysilicon and metal layers when a high current beyond the standard CMOS limit is passed through the polysilicon CMOS-MEMS microheaters for the high-temperature CNT growth process. The microheater characterization challenges were also resolved by the latest generation CMOS chip design. Our accomplished results of local CNT synthesis on CMOS-MEMS structures in CMOS chips can be transferred to a wafer-level process, which shows potential for the commercial manufacturing of compact CNT-based smart sensors.

## Materials and Methods

### Post-processing of the CMOS chips

A standard CMOS chip includes a silicon substrate, dielectric layers for electrical isolation, polysilicon layers, interconnecting metal layers, vias connecting the metal/polysilicon layers and passivation layers on the top for protecting the chip surface. The passivation layers of our CMOS chips were selectively removed during the industrial fabrication process in the regions above the polysilicon CNT growth structures. Those regions were assigned with a ‘PAD’ layer during the chip design in the Cadence Virtuoso software tool to avoid the passivation layers. As a result, the challenging post-processing steps for selectively removing the passivation layers at the chip level were not needed.

The post-processing starts with dry etching of the dielectric layer. For the initially designed CMOS chips, the DER-1 and DER-2 etching recipes were used for SiO_2_ etching. DER-1 was performed using a DRIE system (Plasmalab System 100, Oxford Instruments, UK). In DER-1, sulfur hexafluoride (SF_6_) was used for SiO_2_ etching in a Bosch process. In this process, the duration of each etching cycle was 1 minute, followed by a 2 min cooling cycle using 50 sccm Ar at 30 mTorr chamber pressure. Depending on the desired etching thickness, the number of cycles of this loop was decided. The recipe ends with a final 10-minute cooling cycle using 50 sccm O_2_. DER-2 was performed in an ICP-RIE system (Mini-lock Phantom III, Trion Technology, USA). In this system, CHF_3_ and CF_4_ were used for etching instead of SF_6_-based chemistry. The ICP source was enabled with 200 W power and added in a SiO_2_ RIE recipe^48^ together with an introduction of a 50 sccm Ar cooling cycle to form DER-2. Each etching cycle was 10 min long followed by 5 min of cooling. Similar to DER-1, O_2_ was initially included in DER-2 to increase the etch rate; however, it was later removed as introducing O_2_ also increases silicon etching, thus reducing selectivity.

For the succeeding CMOS chips with improved designs, all dry etchings were performed in another ICP-RIE system (PlasmaPro 100 Estrelas, Oxford Instruments, UK). The developed recipes for this system, DER-3 & DER-4, use CHF_3_ as the primary reactive gas. In these recipes, the RF power is balanced by considering the etch rate, selectivity, and etched surface profile. Higher RF power contributes to raising the ion energy of the gases, which increases the etch rate but can cause damage to the etched surface due to induced physical etching^49^ and reduced selectivity. Higher ICP power corresponds to a higher etch rate due to the increase in ion density of the plasma^50^ and can improve etching with a high aspect ratio^51^. In ICP-RIE, a high aspect ratio and high etch rate are achieved without significant surface damage due to decoupled plasma density and energy^49^; we used reduced RF and high ICP power in DER-3 to ensure that. As we achieved uniform etching with a high aspect ratio at 800 W, the ICP power was not increased further. Moreover, too high ICP power and hence too high plasma density can decrease the etch rate due to a reduced mean free path, causing higher ion collisions in the plasma^50^. The duration of each etching cycle in DER-3 is 5 min, followed by a 38 sccm Ar cooling cycle for 2 min. We repeated the loop 8 times for a total of 40 min of SiO_2_ etching of the CMOS chips. As DER-3 has less selectivity, further etching was continued with DER-4. The ICP source is disabled in DER-4, while higher RF power is used. DER-4 produces a significantly lower etch rate but has the best selectivity among the four recipes. The argon flow rate is increased in this recipe to somewhat improve the etch rate. In this ICP-RIE system, the substrate temperature of the sample holder was set to 0 °C for both DER-3 & DER-4, which results in a relatively higher etch rate compared to 20 °C. Additionally, the low chamber pressures used in the recipes ensure fewer ion collisions and high directionality of etching. The etching and cooling duration of each cycle in DER-4 is the same as that for DER-3. The total required etch period using DER-4 varied from chip to chip, as the thicknesses of the dielectric and polysilicon layers in the CMOS chips differ by tens of nanometres. In most cases, 20–25 min of etching with DER-4 was sufficient for removing the oxide layers to reveal the polysilicon layers and silicon substrate. All SiO_2_ dry etching recipes used are summarized in Table 1.

**Table 1:** Summary of SiO_2_ dry etching recipes used

Dry Etching Recipe	Gases with Flow Rates (sccm)	RF & ICP Power (W)	Pressure (mTorr)	Etch Rate (nm/min)
DER-1	H_2_: 15, SF_6_: 50, O_2_: 5	RF: 50, ICP: 1500	30	~ 100
DER-2	Ar: 30, CHF_3_: 30, CF_4_: 30	RF: 500, ICP: 200	100	~ 90
DER-3	Ar: 38, CHF_3_: 12	RF: 80, ICP: 800	10	~ 105
DER-4	Ar: 95, CHF_3_: 15	RF: 300, ICP: 0	20	~ 30

For isotropic SiO_2_ etching, a typical BOE recipe^44^ was initially attempted before moving towards a more aluminium-friendly etchant. Pad etch is an ammonium fluoride (NH_4_F)-based oxide etchant conventionally used to save aluminium pads, even though it still slowly etches aluminium and can make the metal surface rough, especially when the sample is exposed longer in the etching solvent. The aluminium surface is mostly attacked in the first few minutes before being passivated^43^ by aluminium acetate salt^52^ formed from the acetic acid (CH_3_COOH) in the etchant that protects aluminium from further significant etching. The etchant solution is completed with propylene or ethylene glycol and water. Surfactants are also often used to lower surface tension and increase wetting^53^ in the pad-etch solution. In our version of the recipe, we used ethylene glycol without any surfactant. The duration of this wet etching depends on the required amount of partial microheater underetching. From our experiments, approximately 30 min of etching with Pad-etch resulted in near 400 nm SiO_2_ removal from underneath each side of the exposed polysilicon structures. It is important to note that etching with this etchant should be done in one step to avoid further damaging the aluminium surface. Cleaning the sample after removing it from the etchant removes the passivation layer over the aluminium surface; hence, putting the sample back into the etchant for additional SiO_2_ etching causes more harm to the unprotected metal.

### Characterization of the CMOS chips

The surface of the etched CMOS chips was characterized by two profilometers (P-7 Stylus Contact Profiler, KLA-Tencor, USA & Dektak 150 Surface Profiler, Veeco, USA) with stylus radii from 2 to 12.5 µm. The initially designed CMOS chip has a few dummy polysilicon structures that are large enough for suitable measurements of the poly-1 and poly-2 layers. In the succeeding CMOS chip design, some large metal and polysilicon microstructures are placed in more convenient locations for the purpose of this characterization. After completing the dry etching processes, profiling the polysilicon structures ensures whether they are fully uncovered without experiencing any etching, as shown in Fig. 6b.

A probe station (450PM Manual Probe Station, Micromanipulator, USA) was used to confirm whether all exposed SiO_2_ was etched by measuring the electrical conductivity of the chip surface. The probe positioners/manipulators holding the probes are adjusted to place the probe tips over the polysilicon layers or bulk silicon. A properly etched chip surface shows finite resistance, while even a thin layer of remaining SiO_2_ results in a nonconducting surface in this measurement. Probe tips of ~20–25 µm in diameter were used in this task. Microprobes with thinner tips (1 µm & 3.5 µm) were also tried to examine the smaller polysilicon structures, but those soft tips did not provide good contacts for proper measurement. The probe station was also handy for testing the resistance of some polysilicon microheaters before wire bonding the sample in a chip carrier. A significantly higher heater resistance is found if the polysilicon layer is etched during the ICP-RIE process.

For thermal characterization, infrared microscopy was attempted on the previous generation CMOS chips using an advanced IR camera (A6750 MWIR, FLIR, USA) with a 4X microscopic lens. Although accurate microheater temperatures were not determined due to fundamental resolution limitations, the results indicated a high thermal gradient around the heaters^41^. Thermal characterization can be done more accurately on the latest generation CMOS chips due to the inclusion of the larger microstructures.

Two different SEMs are used for the characterization of the CMOS chips and synthesized CNTs. General characterizations including required energy dispersive X-ray spectroscopy (EDX) analysis were performed in an SEM with a thermionic emission source (SU3500, Hitachi, Japan). A high-resolution field emission SEM (SU8230, Hitachi, Japan) was used for imaging the CNTs and cross-sectional chip characterizations. The characterization is mostly performed at moderate accelerating voltage (5 kV) using a secondary electron detector.

### CNT growth process on CMOS-MEMS heaters

A thin catalyst layer (~3 nm iron) is deposited on the CMOS chips by e-beam evaporation using a multitechnique thin film deposition system (ATC 2030-HY, AJA International, USA). A CMOS chip is then attached to a chip carrier, and the selected contact pads are wire bonded by Au ball bonding using a semiautomatic wire bonding system (5610, F&K Delvotec, Austria). After wire bonding, the chip carrier is configured inside the custom-built CVD chamber. The chamber is evacuated with a vacuum pump until a pressure of ~6–7 mbar is reached. Argon is then supplied to the chamber as a supporting gas with a 100 sccm flow rate. The electrically connected CMOS MEMS microheater temperature is gradually increased to the CNT synthesis temperature (~650–800 °C) by Joule heating in an argon-occupied environment with ~400 mbar chamber pressure. CNTs start to grow on the microheater when a hydrocarbon precursor gas (acetylene) is introduced in the chamber at a 50 sccm flow rate using a mass flow controller (MFC). The CNT synthesis process was carried out for 5–10 min before introducing vacuum and reducing the microheater temperature. Additional steps are performed to establish CNT connections between the microheater and the adjacent microstructure for gas sensing applications^47^.
